# Unbiased assessment of 2-arachidonoylglycerol in cardiovascular inflammation

**DOI:** 10.1038/s41598-025-28969-5

**Published:** 2025-11-21

**Authors:** Moritz Nöthel, Franziska Dorer, Alexandru Odainic, Carolyn Krause, Marie Rüthing, Jasper Spitzer, Ulrike Strube, Theresia Weise, Dieter Lütjohann, Georg Nickenig, Susanne V. Schmidt, Julian Jehle

**Affiliations:** 1 Department of Internal Medicine II, Cardiology, Pneumology, Angiology, University Hospital Bonn, Venusberg-Campus 1, Building 13, 53127 Bonn, Germany; 2 Medical Faculty, Institute of Innate Immunity, University of Bonn, 53127 Bonn, NRW Germany; 3 Department of Microbiology and Immunology, The Peter Doherty Institute for Infection and Immunity, University of Melbourne, Melbourne, VIC 3000 Australia; 4 Institute of Clinical Chemistry and Clinical Pharmacology, University Hospital Bonn, 53127 Bonn, Germany

**Keywords:** 2-arachidonoylglycerol, Endocannabinoid system, Monocytes, Inflammation, Coronary heart disease, Acute coronary syndrome, Biomarkers, Cardiology, Diseases, Immunology

## Abstract

2-arachidonoylglycerol (2-AG) is an endogenous modulator of inflammatory processes. Recent studies demonstrate its key role in inflammatory cardiovascular diseases, such as atherosclerosis and left ventricular remodelling. However, whether 2-AG acts as a pro- or anti-inflammatory agent in cardiovascular inflammation is a matter of controversial debate. CD14^+^ primary human monocytes were treated with 2-AG. Differentially expressed genes were determined by mRNA sequencing and used for bioinformatics analysis of changes in myeloid cell functions and cell signalling pathways. Additionally, we quantified cytokines and mediators involved in cardiac remodelling by multiplexing assays. Human monocytes responded to the treatment with 2-AG by inducing a pro-inflammatory cellular program. The top upregulated hallmark pathways were TNFα signalling via the NF-κB pathway, the inflammatory response and IL-6 signalling, illustrating the pro-inflammatory mode of action of 2-AG. These data were validated at the protein level in the supernatants of stimulated cells using multiplexing assays. Based on these in-vitro results, we examined blood samples from patients during acute and chronic coronary syndromes. By sampling blood from both the periphery and the culprit coronary artery, we observed a trend towards higher local concentrations of the cardiac remodelling factors Galectin-3, Osteopontin, and IP-10 in the coronary arteries of patients presenting with acute coronary syndrome compared with other groups. The levels of these remodelling factors correlate positively with 2-AG levels. Taken together, our data suggest that 2-AG acts as a pro-inflammatory agent in myocardial infarction and LV remodelling.

**Supplementary Information:**

The online version contains supplementary material available at 10.1038/s41598-025-28969-5.

## Introduction

Monocytes represent the predominant non-granulocytic myeloid subset in peripheral blood, accounting for approximately 5–10% of total circulating leukocytes^1,2^. After myocardial infarction, monocytes are recruited to the heart where they differentiate into macrophages^3^. As macrophages fulfil a broad variety of functions in dependency of signals from microenvironment^4^, they can either contribute to inflammation of cardiac tissue or contribute to wound healing and tissue remodelling^5^. Acute myocardial infarction and heart failure are associated with inflammatory immune reactions^6,7^. If the inflammatory immune response is not timely terminated, patients with cardiac injury are more prone to develop adverse remodelling^6,8^. In a murine model for myocardial infarction, prolonged inflammatory immune responses promoted impaired infarct healing and increased susceptibility to cardiac rupture^9^. Likewise in humans, the Interleukin (IL)-1 receptor antagonist anakinra has been shown to suppress the acute inflammatory response after myocardial infarction and inhibit adverse left ventricular (LV) remodelling^10,11^.

In contrast to their pro-inflammatory properties, immune cells can also adopt anti-inflammatory phenotypes to promote myocardial healing after infarction. This involves removing cellular debris, preventing necrotic tissue formation and promoting angiogenesis and scar formation. Several cytokines like e.g. IL-2 and TGFβ are secreted by T_H_2 cells, Treg cells and alternatively activated macrophages to support myocardial wound healing (summarized by^12^).

The endocannabinoid system (ECS) modulates inflammatory processes. The two principal receptors of the ECS are the CB1 receptor (CB1) and the CB2 receptor (CB2), both of which are G protein-coupled cannabinoid receptors. The CB1 receptor is primarily expressed in the central nervous system, particularly at the terminals of central and peripheral neurons, where it mediates the release of various neurotransmitters involved in perception, motor function, and pain perception^13,14^. The CB2 receptor is predominantly expressed in immune cells, where it regulates various immune processes such as migration of peripheral monocytes, neutrophils, eosinophils and NK cells^13^. The endocannabinoids N-arachidonoylethanolamide (anandamide, AEA) and 2-arachidonoylglycerol (2-AG) are the endogenous ligands of the cannabinoid receptors. 2-AG is an endogenous CB1 receptor and CB2 receptor agonist with the strongest stimulatory effect on CB2^13,15,16^.

While earlier studies emphasized the anti-inflammatory effect of 2-AG via the CB2, there is now increasing evidence of its pro-inflammatory effects in cardiovascular disease^17,18^. Schloss et al. recently showed in a murine model for myocardial infarction that 2-AG increases the size of the affected myocardial region and promotes adverse cardiac remodelling^19^. Likewise, in humans, plasma concentrations of 2-AG correlate with the severity of coronary artery disease (CAD), as we could demonstrate in an earlier study^20^. However, data on the interrelation of 2-AG and adverse cardiac remodelling in humans are missing, which encouraged us to further analyse the collected samples. However, the question of whether 2-AG acts as a pro- or anti-inflammatory agent in cardiovascular inflammation and myocardial remodelling remains controversial.

In the present study, we sought to overcome conflicting evidence on the role of 2-AG in cardiovascular inflammation, and to further elucidate its molecular mechanisms in human pathology^21–23^. Therefore, we chose an unbiased approach by stimulating CD14^+^ monocytes from healthy donors with 2-AG, assessing transcriptional changes and cytokine production. We then validated our findings in a cohort of patients with CAD and mapped local concentration profiles of cardiac remodelling factors (CRFs). Our data suggest that 2-AG acts as a pro-inflammatory agent in myocardial infarction and LV remodelling.

## Material and methods

### Isolation of CD14^+^ monocytes

Buffy coats from healthy donors were obtained according to protocols accepted by the institutional review board at the University of Bonn (local ethics votes no. 105/17). Leukocyte separation was performed using a Ficoll gradient (Ficoll Paque, Cytiva, Marlborough, USA) followed by enrichment of monocytes with CD14 magnetic beads (Miltenyi Biotec, Bergisch Gladbach, Germany) and a subsequent positive selection was performed using LS columns (Miltenyi Biotec). The remaining monocytes were dissolved in RPMI 1640 (Thermo Fisher Scientific, Waltham, USA), supplemented with 10% FCS (Thermo Fisher Scientific) and 1% Pen/Strep (Thermo Fisher Scientific), and distributed into a 12-well plate at a final concentration of 6 × 10^5^ cells/ 1.2 ml. The cells were subsequently stimulated with either DMSO (AppliChem, Darmstadt, Germany), or 2-AG [1 µM] (Tocris, Bristol, UK), and cultivated at 37 °C.

After 4 h and 24 h, cells were harvested and dissolved in Trizol (Thermo Fisher Scientific) for further RNA isolation. After 24 h, the supernatants were collected after the depletion of cellular fractions by a short centrifugation step (210 × *g*, 5 min, 4 °C). Supernatants were stored at −80 °C until further use.

### Flow cytometry

2-AG treated monocytes and respective controls were detached using 2 mM EDTA in PBS at room temperature. After an incubation period of 10 min monocytes were transferred into tubes, and centrifuged at 2100 × *g* for 5 min at 4 °C. Sample were resuspended in blocking buffer (2% FCS in PBS) and incubated in the presence of the following antibodies against monocytic markers and CB receptors for 20 min at room temperature in the dark: CD11c (Biolegend, San Diego, USA, clone 3.9), CD14 (Biolegend, clone 63D3), HLA-DR (Biolegend, clone L243), CB1 (LS Bio, LS‑C662519), CB2 (Abcam, ab3561), and FITC donkey anti-rabbit IgG (Biolegend, Poly4064). Cells were washed with 1 mL PBS and centrifuged at 500 × *g* for 5 min at 4 °C. The pellets were resuspended in 600 µL PBS and analysed by an Attune NxT (ThermoFisher) followed by statistical analysis and visualization by FlowJo (v10.9.0).

### RNA isolation

After removing the supernatants, 200,000 monocytes were lysed in 0.7 mL Trizol (Invitrogen/Thermo Fisher Scientific). RNA was isolated according the manufracturer’s recommendations with the RNeasy Micro kit (Qiagen), Venlo, NL). RNA quality was verified by Tapestation 2200 (Agilent, Santa Clara, USA). Total mRNA concentrations were adjusted with RNAse/DNAse free water (Gibco/Thermo Fisher Scientific) to a final concentration of 14 ng/µl for use in mRNA sequencing.

### RNA sequencing

To determine transcriptomic changes between 2-AG-activated and untreated monocytes, 3′-mRNA sequencing was performed with up to 56 ng purified RNA (NGS Core Facility, University Hospital Bonn, Germany). The library production was performed according to the manufacturer’s protocol and sequenced on a HiSeq2500 (Illumina, San Diego, USA) at the (NGS Core Facility, University Hospital Bonn, Germany) with a sequencing depth of 10mio reads per sample.

### Bioinformatics analysis

Reads were aligned with STAR (v2.7.8a) against the human reference genome hg19. Transcripts were quantified with the Partek E/M algorithm and further processed for normalization in R with the DEseq2 algorithm. Batch effects derived from donor-specific differences were removed in the Partek Genomics Suite (v7.20.0831). The dataset was further optimized by flooring transcripts with minimal gene counts at least to 1 and the exclusion of transcripts with a mean expression below 10 counts in every test condition. Differentially expressed genes between 2-AG-activated and unstimulated monocytes were determined by a two-way-ANOVA (fold-change |1.5|, FDR-adjusted p value < 0.05). Data visualization and biological interpretation were performed with the Partek Genomics Suite and R (v4.3.1) packages ggplot2 (v3.4.2) for graphical visualization of expression data and tidyr (v1.3.0) for data wrangling. Statistical analysis was performed using the rstatix package (v0.7.2) and visualized using the ggpubr package (v.0.6.0).

Enrichment of genes associated to 2-AG expression was performed on a ranked expression list using the GSEA function from the clusterProfiler package (v4.8.2;^24^).

### Quantification of cytokines in cell culture supernatants

Cytokines in the supernatants were quantified by multiplex analysis (CorPlex, 116-7Bf-1-AB, Quanterix, Billerica, USA), according to the manufacturer’s protocol. Briefly, the thawed samples were mixed thoroughly and centrifuged (10,000 × *g*, 4 °C, 5 min). The supernatant was collected and diluted 1:4 with the sample diluent.

The calibrator was then prepared in a serial dilution (1:1 to 1:4096). Before application, the plate was washed using the Simoa Microplate Washer (Quanterix). Subsequently, the samples and the pre-diluted calibrator were transferred to the wells and incubated for 2 h (room temperature, shaken at 525 rpm). Afterwards the samples were washed, the residual wash buffer was removed completely and 50 µl of the biotinylated antibody reagent was added. After another 30 min of incubation (room temperature, shaken at 525 rpm), the samples were washed again, streptavidin-HRP reagent was added and then incubated for 30 min (room temperature, shaken at 525 rpm). Finally, the plate was washed one more time using the SP-X post HRP Wash 2.0 protocol, the SuperSignal substrate was added and the plate was immediately read on an SP-X imaging system (Quanterix).

### Collection of blood samples from patients undergoing cardiac catheterization

Blood samples were drawn from patients undergoing coronary angiography for suspected or known coronary artery disease. Blood samples were collected from the sheath, the aortic sinus and the post-stenotic coronary artery. Samples were collected and endocannabinoid concentrations were quantified as part of an earlier study^20^. Written informed consent was obtained from all patients. The study protocol was approved by the local ethics committee of the University Hospital Bonn (Ethics vote no. 306/15). All protocols adhered to the Declaration of Helsinki and its subsequent revisions. In- and exclusion criteria, as well as the detailed sampling protocol are published^20^. The access route was chosen independently by the investigator: either radial or femoral using an arterial sheath (Terumo 6F; Terumo, Tokyo, Japan). Blood samples were drawn from the aortic sinus using a pigtail catheter (Boston Scientific, Marlborogh, USA). Samples from the coronary circulation were taken from the poststenotic coronary artery using an over-the-wire catheter (1.5 mm /10 mm OTW balloon catheter, Boston Scientific) prior to angioplasty.

### Quantification of protein markers of left ventricular remodelling in human plasma samples

In human plasma samples, the levels of proteins that are involved in cardiac remodelling were determined by a bead-based multiplex ProcartaPlex assay (Thermo Fisher). The assay was performed as recommended by the manufacturer.

Cytokine measurement was performed with the Luminex™ FLEXMAP 3D™ Instrument System (Thermo Fisher Scientific).

### Statistical analyses

Data were analyzed using Microsoft Excel (Microsoft, Redmond, USA), SigmaPlot (Grafiti LLC, Palo Alto, USA) and GraphPad Prism software (GraphPad Software, San Diego, USA). For the number of healthy donors, a sample size calculation was performed using the program G*Power. Assuming an effect size of 1.2 and an α error of 0.05, a total sample size of 8 per group was found to be necessary for an actual power of 0.8.

For paired data, a two-tailed paired t-test was used to calculate p values. In case the normality test failed, a Wilcoxon Signed Rank Test was performed. Adjusted p values less than 0.05 were considered statistically significant. Deviations are outlined in figure legends.

## Results

### 2-AG leads to transcriptomic changes in monocytes and activates pro-inflammatory signalling pathways

**Fig. 1:**
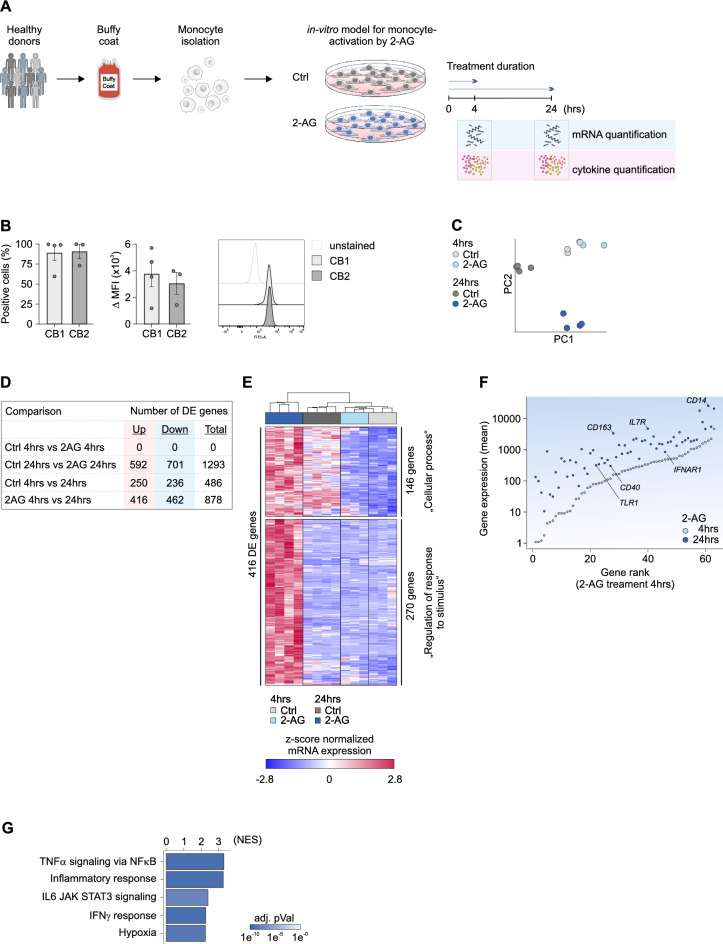
2-AG induces transcriptomic changes in human monocytes with pro-inflammatory features. (**A**) Shema of in vitro culture of human monocytes isolated from buffy coats of healthy donors. Monocytes are cultured in presence and absence of 2-AG for up to 24 h. (**B**) Bar charts of flow cytometry analysis of the endocannabinoid receptors CB1 and CB2 on human monocytes which serve as receptors for 2-AG. (**C**) Principle component analysis of the transcriptomes of monocytes treated with 2-AG and respective controls. (**D**) Overview on the number of differentially expressed (DE) genes identified by 2way-ANOVA with a fold-change of |1.5| and p-Value < 0.05. (**E**) Heatmap of 416 genes which were significantly up-regulated in its expression by 2-AG. Genes were categorized in to clusters according their GO (gene ontology) term categorization. (**F**) Dot plot for the 270 2-AG induced genes belonging to the GO term “Regulation of response to stimulus”. Gene of the top 2-AG induced surface markers are marked in the plot. (**G**) Bar chart of the top 5 enriched hallmark gene sets in transcriptomic data sets of human monocytes cultured for 24 h in the presence of 2-AG compared to monocytes cultured for 24 h without 2-AG. (n = 3–4).

We treated human peripheral monocytes with 2-AG for up to 24 h to investigate transcriptomic changes and potential changes in monocyte functions (Fig. 1A and Supplementary Fig. 1A). Initially, we validated the expression of the cannabinoid receptors CB1 and CB2 by monocytes via flow cytometry (Fig. 1B, Supplementary Table 1). The majority of monocytes showed a strong expression of CB1 (89 ± 10%; MFI: 3.8 × 10^3^ ± 0.9 × 10^3^) and CB2 (91 ± 8%; MFI: 3.0 × 10^3^ ± 1.0 × 10^3^). Based on this result, we concluded that monocytes are susceptible to 2-AG stimulation.

Flow cytometry analysis after prolonged stimulation with 2-AG for up to 24 h showed that neither the percentage of CB1- and CB2-positive cells nor the mean fluorescence intensity changed significantly (Supplementary Fig. 1B).

RNA sequencing technology was used to investigate the effects on myeloid cell programming by 2-AG treatment (Supplementary Table 2). These analyses show the induction of a pro-inflammatory phenotype in 2-AG-treated monocytes:

Overall, the transcriptomes of monocytes left untreated (Ctrl) and 2-AG-stimulated were comparable after 4 h of cell culture as we were not able to detect any differentially expressed (DE) genes between both groups (Fig. 1C,D; Supplementary Table 3). However, the number of DE genes increased with the duration of cell culture (Fig. 1D). Gene ontology (GO) analysis for a total number of 486 genes, which changed in their expression significantly over the period of cell culture in absence of 2-AG, were focused on the metabolism (Supplementary Fig. 1C). Prolonged (24 h) culture of monocytes in presence of 2-AG led to the regulation of 878 genes (Fig. 1D). The group of 462 genes which were suppressed after 24 h of 2-AG treatment were categorized as metabolism related (Supplementary Fig. 1D). The group 2-AG induced genes (Fig. 1E) could be further categorized into a group of 146 genes, which associated to the GO term “cellular process” and showed slightly elevated expression levels after 24 h of culture in absence of 2-AG. Interestingly, there was another set of 270 DE genes, which were significantly upregulated in the presence of 2-AG after 24 h. Those genes were associated to biological functions like “regulation of response to stimulus”. In total, 63 genes out of this distinct group (23%; pVal = 1.4 × 10^–8^, Fisher’s exact test; Fig. 1F) belong to the surfaceome, which displays an over-representation of surface markers with respect to the distribution of surfaceome associated genes in the whole data set (13%). Surface markers of myeloid cell functions, like *CD40*, *CD163*, *IL7R*, *TRL1*, *IFNAR1* and *CD14* were amongst the 2-AG induced surface markers. In consequence, increased surface marker expression might render 2-AG treated monocytes into a highly sensitive state with subsequent elevated states of signal integration and intracellular signalling pathways. One of the top-induced genes was *CD14*. The CD14 receptor and its collaborating Toll-like receptor 4 (TLR4) is involved in recognition of bacterial compounds. Downstream signalling via the adaptor molecule MyD88 leads to the activation of the transcription factors NfkB and IRF3 which participate in the transcription of pro-inflammatory mediators like TNFα, IL1β and interferons^25^.

The interpretation of mRNA seq data sets can be influenced by the usage of hard cut offs and fold-changes (FCs). Both parameters are configured by the analyst and represent a form of analytical bias. Therefore, we extended our data mining to hallmark analysis. This approach enables the identification of biological functions in immune cells based on the integration of the complete gene expression profile. After 24 h of culture, 2-AG induced genes which participate in “TNFα signalling via the NF-κB” (NES) = 3.3, pVal < 1 × 10^–9^), “Inflammatory response” (NES = 3.3; pVal < 1 × 10^–9^), “IL6 JAK STAT3 signalling” (NES = 2.4; pVal < 3.8^–8^), and “INF-γ response” (NES = 2.3; pVal < 1 × 10^–9^).

In summary, our transcriptome analysis of 2-AG-treated monocytes indicates the induction of a pro-inflammatory phenotype and an elevated state of signalling potential in a time-dependent manner.

### 2-AG promotes cytokine release from human monocytes

**Fig. 2:**
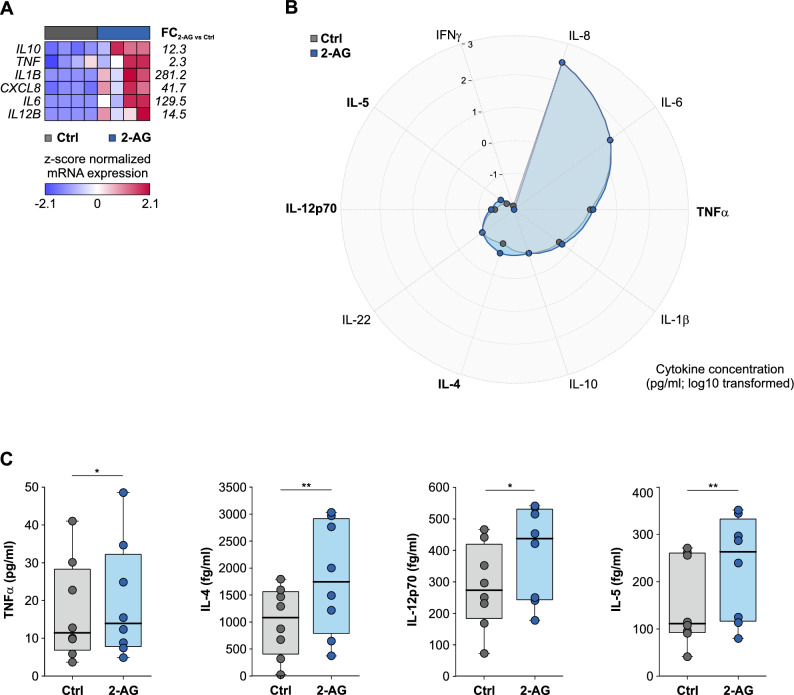
Human monocytes release pro-inflammatory cytokines after 2-AG treatment. Peripheral human CD14^+^ monocytes were cultured for 24 h in the presence and absence of 2-AG. (**A**) Heatmap for z-score transformed transcript levels of pro-inflammtory cytokines in monocytes activated by 2-AG and respective controls. (**B**) Radar plot for log10-transformed cytokine concentrations in the supernatant of monocytes cultured with or without 2-AG. Cytokines significantly induced by 2-AG treatment are marked by bold letters. (**C**) Box-and-whisker plots for cytokine concentrations which increased significantly after 2-AG treatment of human peripheral monocytes. (n = 8).

Our transcriptome analysis indicated that 2-AG can induce a pro-inflammatory program in human monocytes. In addition to the overexpression of surfaceome-associated genes, we found that the mRNA expression of a large number of immunomodulatory cytokines was differentially regulated: We focused on a set of pro- and anti-inflammatory mediators and observed that transcripts for several cytokines, amongst them IL-6, IL-1B, IL-10, TNFα and IL-12 were increased after stimulation with 2-AG (Fig. 2A).

Following the lead, that 2-AG induces transcriptomic changes of immunomodulatory cytokines, we next investigated whether 2-AG also promotes the production and release of cytokines on the protein level. We used a multiplex assay that best matched our findings on the mRNA level and that covered both pro- and anti-inflammatory cytokines. (Fig. 2B, Supplementary Table 4). We did not observe any significant induction of pro-inflammatory interleukins, like IL-8, IL-6 and IL-1β. Yet, concentrations of three cytokines involved in inflammatory immune responses, namely TNFα, IL-12p70 and IL-5, were significantly elevated in monocytes cultured for 24 h in the presence of 2-AG (TNFα: X̅_Ctrl_ = 17.0 ± 4.6 pg/ml, X̅_2-AG_ = 19.6 ± 5.4 pg/ml, pVal_TNFα_ = 0.012; IL-12p70: X̅_Ctrl_ = 284.9.0 ± 47.2 fg/ml, X̅_2-AG_ = 392.1 ± 52.1 fg/ml; pVal_IL-12p70_ = 0.012; IL-5: X̅_Ctrl_ = 155.0 ± 32.7 pg/ml, X̅_2-AG_ = 229.7 ± 38.5 pg/ml, pVal_IL-5_ = 0.008) (Fig. 2C).

Interestingly, IL-4, which is a representative of the anti-inflammatory cytokines, was also differentially regulated in the supernatants: 2-AG upregulated the secretion of IL-4 in our *in-vitro* model for human peripheral monocytes about 2-times (IL-4: X̅_Ctrl_ = 1003.8 ± 224.7 pg/ml, X̅_2-AG_ = 1811.1 ± 369.6 pg/ml, pVal_IL-4_ = 0.002).

In summary, 2-AG induces a significant up-regulation and release of immunomodulatory cytokines in human monocytes. These include predominantly, but not exclusively pro-inflammatory cytokines. Given that all these cytokines (pro- and anti-inflammatory) have been associated with immunological processes following myocardial infarction and during left ventricular remodelling, we next sought to elucidate, if there is a connection between 2-AG-induced cytokine production and hallmark effectors of myocardial remodelling.

### Biomarkers of left ventricular remodelling correlate with 2-AG in humans

First, we measured protein concentrations of eight CRFs in the supernatant of human CD14^+^ monocytes in vitro after stimulation with 2-AG*.* We found the protein levels of all eight CRFs unchanged after the stimulation with 2-AG (data not shown). However, this experiment may not adequately reflect the processes involved in myocardial remodelling, as remodelling is a complex process that is influenced not only by monocytes but also by a variety of different cell types. Previously, we reported that 2-AG levels increase with disease progression in CAD- and Non-ST-segment elevation myocardial infarction (NSTEMI) patients, and that 2-AG concentrations vary within the human arterial vasculature, with peak concentrations measured in the coronary arteries during NSTEMI^20^. These concentration gradients may create a distinct microenvironment within the myocardium that promotes cardiac remodelling.

**Fig. 3:**
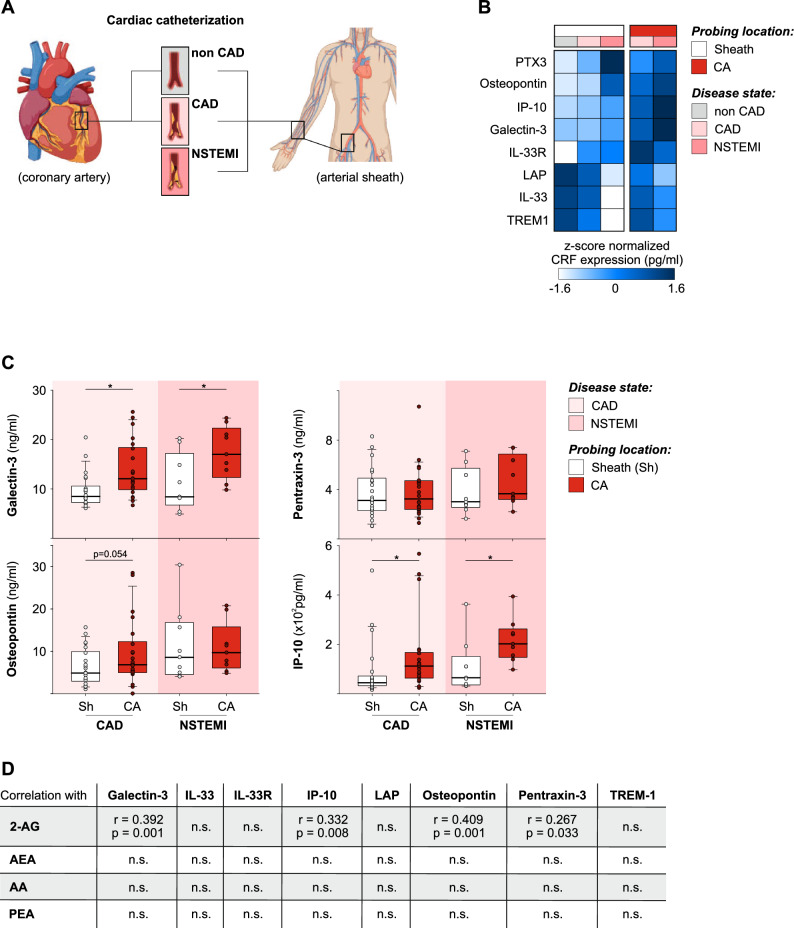
Expression of CRFs increases with disease progression of cardiac diseases. (**A**) Schema of blood sample acquisition and overview on patient groups undergoing cardiac catheterization. (**B**) Heatmap of Z-score transformed CRF concentrations in CAD and NSTEMI patients as well as control patients (non CAD). Samples were taken from the periphery (Sheath, Sh) or the cardiac aorta (CA). (C) Box-and-whisker plots for CRFs which increase with disease severity and maximum plasma concentrations in the CA. (**D**) Statistics for the correlation of CRFs with 2-AG levels in the sheath were calculated as Pearson’s correlation coefficients (r). (n = 9–42).

To investigate, if CRFs correlate with 2-AG concentrations in CAD- and NSTEMI-patients, we analyzed blood samples from a patient cohort undergoing cardiac catheterization (Fig. 3A). Blood samples were collected during an earlier study^20^ at the coronary artery itself and at the sites of the arterial sheath at the radial or femoral artery. Patients were staged according to their diagnosis into three groups: non CAD, CAD and NSTEMI. Plasma samples were screened for eight CRFs (Fig. 3B, Supplementary Table 5). Z-score transformed concentrations of CRFs in the peripheral blood from the sheath like Pentraxin-3, Osteopontin, IP-10, Galectin-3 and IL-33R increased with the disease severity. For example, the concentrations for PTX3 increased slightly (Sheath concentrations for PTX3: X̅_nonCAD_ = 3126 ± 476 pg/ml, X̅_CAD_ = 3627 ± 348 pg/ml, X̅_NSTEMI_ = 5374 ± 957 pg/ml) in CAD patients FC_nonCAD vs CAD_ = 1.2 × and increased even more in NSTEMI patients (FC_nonCAD vs NSTEMI_ = 1.8x) in comparison to patients without CAD. Moreover, plasma concentrations of Galectin-3, Osteopontin, Pentraxin-3 (PTX3) and IP-10 were even higher in the coronary artery with maximum concentrations in NSTEMI patients (Fig. 3C). For example, Galectin-3 levels in the coronary artery were up to 1.5-times higher in comparison to the respective concentrations in the periphery (CAD: X̅_Sheath_ = 9873 ± 469 pg/ml, X̅_CA_ = 13993 ± 1140 pg/ml; NSTEMI: X̅_Sheath_ = 11487 ± 1333 pg/ml, X̅_CA_ = 17337 ± 1775 pg/ml). Overall, Galectin-3 and Osteopontin displayed the highest CRF concentrations in plasma of those cytokines which increased with disease severity, followed by PTX3 and IP-10.

Finally, we analyzed the correlation between 2-AG and the concentrations of remodeling markers by pearson correlation analysis (Fig. 3D). This analysis revealed significant positive correlations between 2-AG and Galectin-3 (r = 0.39; p = 0.001), 2-AG and IP-10 (r = 0.33; p = 0.008), 2-AG and Osteopontin (r = 0.41; p < 0.001), and 2-AG and PTX3 (r = 0.27; p = 0.033).

**Fig. 4:**
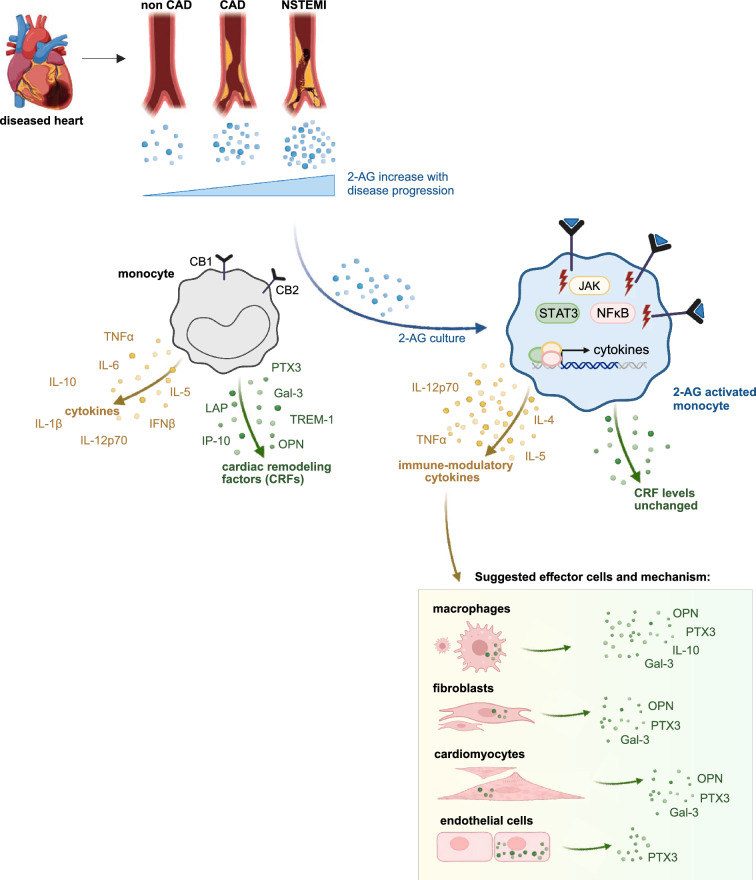
Schema of inflammatory circuits induced by 2-AG in cardiac diseases. Concentrations of 2-AG correlate with the severity of coronary artery disease. Upon 2-AG treatment, immune signaling cascades lead to an enhanced expression of immune-modulatory cytokines like TNFα via JAK, STAT3 and NFκB. Hypothetically, enhanced levels of released cytokines have a systemic effect but also local effects on resident cells of the cardiac tissue, like macrophages, cardiomyocytes, fibroblasts and endothelial cells. With disease progression this inflammatory circuit is increasingly active and leads to the production of CRFs in cardiac tissue.

In summary, we observed that cardiac remodelling factors PTX3, Osteopontin, IP-10 and Galectin-3 increase with disease severity and correlate positively with 2-AG concentrations (Fig. 4).

## Discussion

For a very long time, the impact and functions of 2-AG in cardiovascular inflammation were discussed controversially and its role in cardiac remodelling was uncertain. Conflicting data showed that 2-AG can have pro- as well as anti-inflammatory effects in coronary heart diseases^19,21,23,26–29^. In order to clarify this controversy, we set out to examine the effects of 2-AG in human monocytes in an unbiased way.

To elucidate the role of 2-AG in cardiovascular inflammation, we used peripheral monocytes isolated from blood of healthy donors and cultured them in the presence of 2-AG for 24 h and performed transcriptomics analysis. RNA-seq analysis revealed that 2-AG activates a powerful pro-inflammatory program in monocytes affecting surface marker expression and immune signalling cascades. Hallmark enrichment analysis suggests that 2-AG activates transcription factors like NFκB, JAK and STAT3 which participate in the transcription of pro-inflammatory cytokines, like TNFα (Fig. 4), which are increasingly released from peripheral monocytes in dependency from 2-AG. Our data demonstrate that 2-AG activates pro-inflammatory programs that drive cardiovascular inflammation^19,21,23,27,28^. The results presented here are consistent with those of earlier studies by our group, which suggested that 2-AG acts as a pro-inflammatory agent in various cell culture and animal models^20,21,28,30–33^. Based on the data from Schloss et al*.* and our own findings, we presume that the changes in mRNA expression mediated by 2-AG are primarily mediated by the CB2 receptor, as this receptor has been demonstrated in former studies to be mainly responsible for the recruitment of myeloid cells and atherogenic processes^19,21^.

Another receptor sensitive to cannabinoid stimulation is GPR55. At least for neutrophils, it has been described that GPR55 is not only targeted by ECS ligands but also regulates CB2 signalling pathways and augments the migratory response^34^. Meta data of publicly available data repositories on mRNA level in different human tissues and cells (www.proteinatlas.org) show, that GPR55 is expressed on T cells and B cells. However, on mRNA level GPR55 is not expressed in our cultured monocytes (data not shown). Therefore, we did not look on the effect of 2-AG on GRP55.

On the translational level, we found that 2-AG promotes the release of various cytokines from monocytes. Interestingly, many of the regulated cytokines play crucial roles in myocardial remodelling:

E.g., TNFα is a known driver of left ventricular remodelling and is associated with adverse outcomes in CAD patients^35^. Activation of TNFα by 2-AG might trigger adverse left ventricular remodelling in CAD and NSTEMI patients. IL-5 is linked to eosinophil-mediated inflammation and might predict outcome after myocardial infarction^36,37^. Secretion of IL-12 is known to aggravate cardiac fibrosis, while IL-12 deficiency reduces cardiac injury after myocardial infarction^38–40^. IL-4 promotes the expression of interstitial collagen content, leading to enhanced myocardial fibrosis and cardiac failure^41,42^.

Intriguingly, an elegant animal study by Schloss et al*.* recently suggested, that 2-AG promotes inflammation and adverse left ventricular remodelling in mice after myocardial infarction^19^. Our data support the findings by Schloss et al*.* and suggest that their murine data might translate into human pathology.

In order to explore the potential mechanistic link between 2-AG-induced inflammation during chronic and acute coronary syndromes and the release of cardiac remodelling factors, we mapped and correlated plasma concentrations of 2-AG and CRFs in patients undergoing cardiac catheterization.

Local concentration peaks of 2-AG and CRFs support the known auto- and paracrine mechanism of action of endocannabinoids. We propose that 2-AG triggers the cytokine secretion from activated monocytes at sites of cardiac injury which then promotes the release of cardiac remodelling factors.

Our data show that the release of the cardiac remodelling markers IP-10, Galectin-3, Osteopontin and Pentraxin-3 increases with CAD severity. IP-10, Galectin-3, and Osteopontin display a local concentration profile with higher concentrations in the coronary circulation than in peripheral blood, which has not been demonstrated before. Intriguingly, the remodelling markers IP-10, Galectin-3, Osteopontin and Pentraxin-3 correlate significantly with plasma concentrations of 2-AG.

The CRFs IP-10, Osteopontin, and Pentraxin-3 are produced by inflammatory cell types like monocytes, macrophages and DCs but also by resident cell types such as cardiomyocytes, cardiac fibroblasts and endothelial cells, as outlined in (Fig. 4). Specifically, macrophages are the predominant source of Osteopontin, Galectin-3, Pentraxin-3 and IP-10. Fibroblasts and cardiomyocytes also contribute to the expression of Osteopontin, galectin-3 and pentraxin-3, while endothelial cells are a source of pentraxin-3^43–51^. Expression and release of those CRFs is activated under inflammatory conditions via TNFα and NF-κB signalling^43,52,53^. Interestingly, we did not observe increased CRF levels in supernatants of 2-AG activated monocytes. This led us to the assumption that there might be an intermediate step involved, most probably involving other cell types like macrophages, endothelial cells, fibroblasts and cardiomyocytes. Therefore, we conclude that TNFα and other cytokines released by 2-AG-activated monocytes lead to the release of CRFs from bystander cells in the injured cardiac tissue, which in consequence lead to adverse left ventricular cardiac remodelling. Therefore, we conclude that TNFα and other cytokines released by 2-AG-activated monocytes lead to the release of CRFs from bystander cells in the injured cardiac tissue, which in consequence lead to adverse left ventricular cardiac remodelling. Via this effect, monocytes and their inflammatory signature are pivotal mediators and regulators in cardiac remodelling.

To reproduce this comprehensively in our in vitro assay, monocytes from the cardiac circulation or macrophages from the infarct site would be of interest. However, as monocytes normally circulate for 1–3 days, we approached this question using circulating monocytes from the periphery (cubical vein) and stimulated them with 2-AG to mimic the local gradient observed in our earlier study^20^. We know from an earlier study that 2-AG has an effect on chemotaxis and monocyte migration, suggesting that these monocytes might account for a part of the latter macrophages^28^. Obviously, a complete modelling system incorporating macrophages and fibroblasts would be of interest for this work. However, we identified monocytes as central mediators of inflammatory signalling in this study, which is in line with our previous studies published for example in the context of atherosclerosis^21,28^. Monocytes are not thought to be primary effector cells, but they nevertheless play a crucial role in the development of disease. We summarized these scientific observations into a suggested mechanistic model in (Fig. 4).

Our study is an example of how the endocannabinoid system orchestrates these inflammatory responses in the context of complex tissues.

In our study, we focused on CD14⁺ monocytes, accounting only for the classical and intermediate monocytes, which together make up more than 90% of the monocyte population^54,55^. Both subgroups are recruited during myocardial infarction and mediate pro- and anti-inflammatory effects^54,56^. However, given the tested hypothesis of the current study, the effects of non-classical monocytes have not been investigated. This limits the scope of the study. Another limitation of the present study is the absence of paired analyses using monocytes from healthy donors and not directly isolated from CAD and NSTEMI patients. This may provide deeper mechanistic understanding in future studies.

## Conclusion

The present study provides unbiased evidence that 2-AG promotes cardiac inflammation via the TNFα and NF-κB related signalling pathways. Correlations and mechanistic data from earlier trials suggest that these inflammatory pathways may trigger the release of IP-10, Galectin-3, Osteopontin and Pentraxin-3, causing left ventricular remodelling in CAD and NSTEMI patients.

## Supplementary Information

Supplementary Information 1.

Supplementary Information 2.

Supplementary Information 3.

Supplementary Information 4.

Supplementary Information 5.

Supplementary Information 6.

## Data Availability

RNAseq data are available on GEO: GSE301847 (reviewer token: glwjgwusrnadfsr).

## Abbreviations

AEA: N-arachidonoylethanolamide, anandamide
2-AG: 2-arachidonoylglycerol
CAD: Coronary artery disease
CB1: Cannabinoid receptor 1
CB2: Cannabinoid receptor 2
CD: Cluster of differentiation
CRFs: Cardiac remodelling factors
eCB: Endocannabinoids
ECS: Endocannabinoid system
IL: Interleukin
IRF3: Interferon regulatory factor 3
IP-10: C-X-C motif chemokine ligand 10 syn. interferon gamma-induced protein 10
MAGL: Monoacylglycerol lipase
NES: Normalized enrichment score
NF-κB: Nuclear factor kappa-light-chain-enhancer of activated B cells
NK cells: Natural killer cells
NSTEMI: Non-ST-segment elevation myocardial infarction
PTX3: Pentraxin-3
TGFβ: Transforming growth factor β
T_H_2: T helper type 2
TLR4: Toll-like receptor 4
TNFα: Tumor necrosis factor α
Treg: Regulatory T cells
