# Supplementary figures and images for: Unbiased assessment of 2-arachidonoylglycerol in cardiovascular inflammation
Source: Sci Rep. 2025 Nov 21;15:41469. doi: 10.1038/s41598-025-28969-5 (PMC12645022; doi:10.1038/s41598-025-28969-5)

Supplementary Figure 1

A

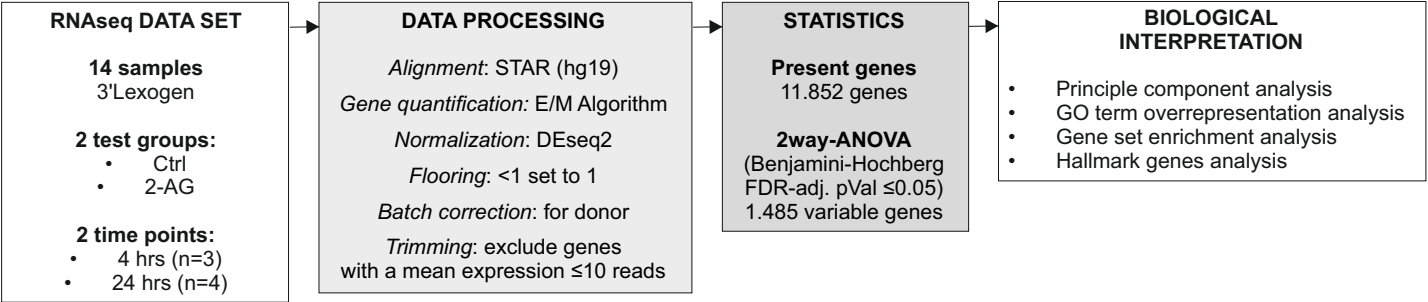

B

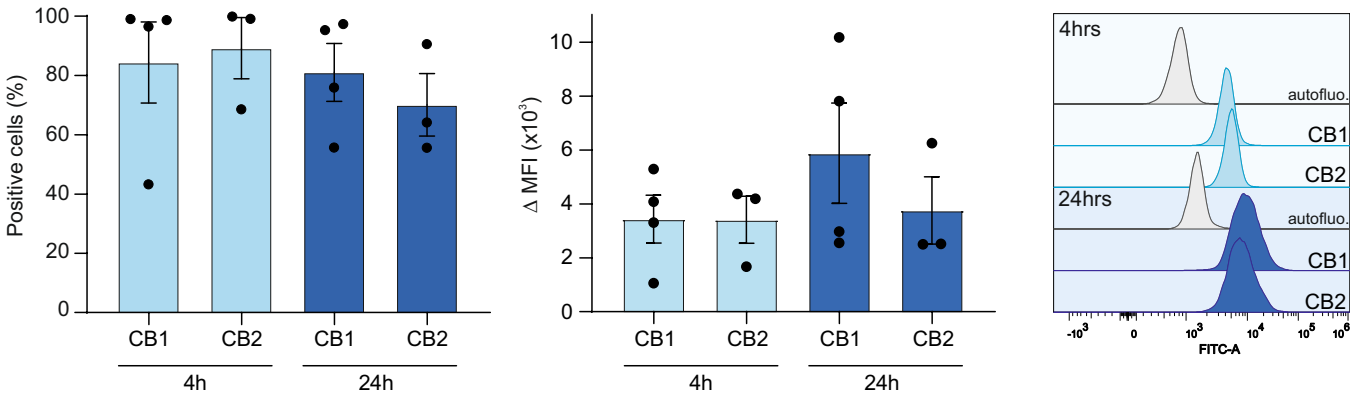

C

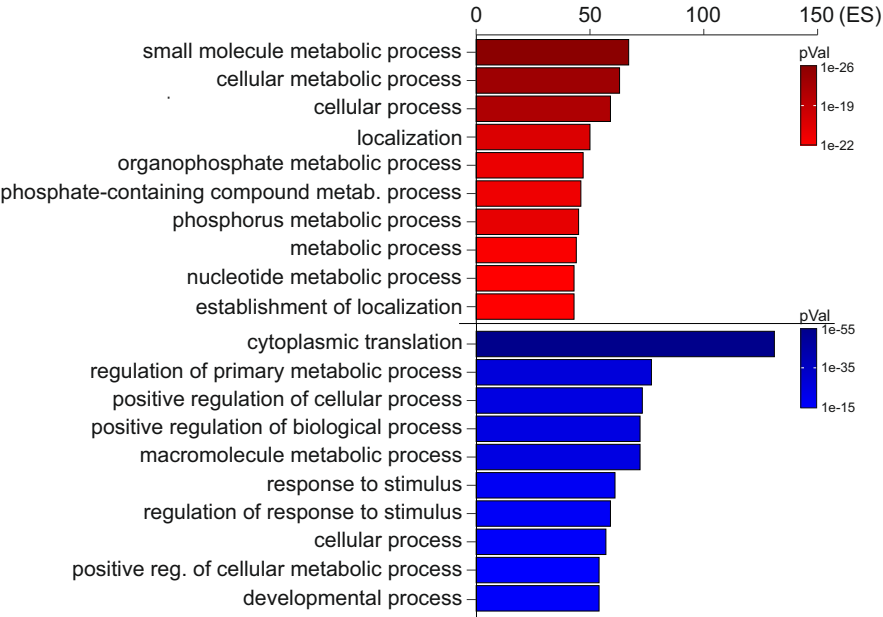

D

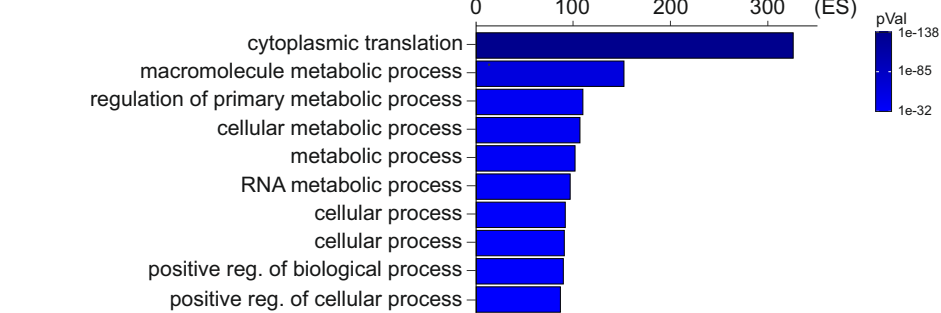

Supplement: Supplementary file 1 — Supplementary Information 1. [file 41598_2025_28969_MOESM1_ESM.pdf]
